# Association of Hearing Loss With Psychological Distress and Utilization of Mental Health Services Among Adults in the United States

**DOI:** 10.1001/jamanetworkopen.2020.10986

**Published:** 2020-07-20

**Authors:** Robin T. Bigelow, Nicholas S. Reed, Katharine K. Brewster, Alison Huang, George Rebok, Bret R. Rutherford, Frank R. Lin

**Affiliations:** 1 Department of Otolaryngology–Head and Neck Surgery, Johns Hopkins University School of Medicine, Baltimore, Maryland; 2 Cochlear Center for Hearing and Public Health, Johns Hopkins University Bloomberg School of Public Health, Baltimore, Maryland; 3 Columbia University College of Physicians and Surgeons, New York State Psychiatric Institute, New York; 4 Department of Mental Health and Center on Aging and Health, Johns Hopkins University, Baltimore, Maryland

## Abstract

**Importance:**

Psychological distress affects health and health care utilization. Hearing loss (HL) is highly prevalent and undertreated, and it may be a potentially modifiable risk factor for psychological distress.

**Objective:**

To investigate the association between HL, psychological distress, and mental health care utilization among adults in the United States.

**Design, Setting, and Participants:**

This cross-sectional study included 25 665 individuals aged 18 years and older who participated in the 2017 National Health Interview Survey, a home-based nationally representative cross-sectional survey of noninstitutionalized US adults conducted by trained interviewers.

**Exposures:**

Self-reported hearing and hearing aid use.

**Main Outcomes and Measures:**

Kessler psychological distress scale (scores ≥5 indicating moderate distress), self-reported use of medication to treat depression or anxiety symptoms, and use of mental health services in the past year.

**Results:**

A total of 25 665 adults (mean [SD] age 47.0 [18.1] years, 51.7% [95% CI, 51.0%-52.5%] women, weighted to be representative of the US adult population) were included in the analysis. Compared with 11 558 participants (49.3%; 95% CI, 48.2%-50.5%) with no HL, the 9390 (35.3%; 95% CI, 34.4%-36.2%) with mild HL and 4717 (15.4%; 95% CI, 14.8%-16.0%) with moderate or worse HL had increased odds of moderate psychological distress (mild HL: odds ratio [OR], 1.49; 95% CI, 1.35-1.62; moderate HL: OR, 2.12; 95% CI, 1.87-2.41) and were more likely to report antidepressant medication use (mild HL: OR, 1.39; 95% CI, 1.17-1.67; moderate HL: OR, 2.07; 95% CI, 1.70-2.57), and antianxiety medication use (mild HL: OR, 1.39; 95% CI, 1.16-1.67; moderate HL: OR, 1.94; 95% CI, 1.57-2.39). Moderate HL was associated with increased odds of use of mental health services (moderate HL: OR, 1.53; 95% CI, 1.30-1.79). Among individuals with moderate HL, those with hearing aids (1066 [22.6%]) were less likely to experience psychological distress than those without hearing aids (3651 [77.4%]; OR, 0.66; 95% CI, 0.53-0.83).

**Conclusions and Relevance:**

In a large nationally representative sample of US adults, self-reported HL was associated with both greater psychological distress and increased rates of antidepressant and antianxiety medication use and utilization of mental health services. Further research is needed to investigate whether HL may be a modifiable risk factor for these outcomes.

## Key Points

**Question:**

What is the association between hearing, hearing aid use, psychological distress, and utilization of mental health services?

**Findings:**

In this cross-sectional study using data from 25 665 participants in the 2017 National Health Interview Survey, hearing loss was associated with increased odds of psychological distress and increased utilization of mental health services. Hearing aid use was associated with decreased odds of psychological distress.

**Meaning:**

The results of this study suggest that hearing loss may be a modifiable risk factor for psychological distress.

## Teaser

This cross-sectional study investigates the association between hearing loss, psychological distress, and mental health care utilization among adults in the United States.

## Introduction

Psychological distress is often characterized by depression and anxiety and is ranked by the World Health Organization as the single largest contributor to global disability.^1,2^ Individuals with psychological distress have increased utilization of mental health services and have overall higher mental health and non–mental health care expenditures.^3,4^ While the causes of psychological distress are complex and multifactorial, recent studies suggest that hearing impairment may be a modifiable risk factor for psychological distress in older adults.^5,6^

Hearing loss (HL) is prevalent in two-thirds of adults older than 70 years^7^ and could contribute to psychological distress.^5^ Several authors have suggested that HL also contributes to depression, possibly mediated by increased social isolation and changes in brain structure related to HL.^5,6^ Importantly, the mechanisms through which HL may affect these outcomes may be modifiable via treatments (ie, hearing aids)^8,9^ that remain vastly underutilized among those with HL.^10^

The existing literature suggests potential associations between HL and psychological distress, but studies have been limited by small sample sizes or nongeneralizable cohorts.^11,12,13,14^ Whether HL could also be associated with mental health care and medication utilization and whether these associations could be modified through the use of hearing aids also remains unknown. In the present study, we analyze data from the 2017 National Health Interview Survey (NHIS) to describe the association between HL, psychological distress, and mental health care utilization and to conduct exploratory analyses to assess the association of hearing aid use with observed outcomes.

## Methods

### Analytic Cohort

The NHIS is an annual cross-sectional household interview survey conducted by the National Center for Health Statistics to evaluate the health of the civilian, noninstitutionalized US population. The survey uses a stratified multistage sample design with oversampling of individuals from minority groups. We used data from the 2017 sample, which consisted of a total of 26 734 adults aged 18 years and older.^15^ Our primary analytic cohort comprised 25 665 individuals with complete data on hearing, covariates, and other outcomes of interest. The NHIS was evaluated and approved by the Research Ethics Review Board of the National Center for Health Statistics. Informed consent was obtained from the respondent to participate in the household interview. This study followed the Strengthening the Reporting of Observational Studies in Epidemiology (STROBE) reporting guideline.

### Hearing Assessment

Sample participants were asked by interviewers to rate the quality of their hearing without a hearing aid. Responses were analytically collapsed into 3 hearing categories, as follows: no HL (response of excellent hearing), mild HL (response of good hearing), and moderate or worse HL (responses of has a little trouble hearing, has moderate trouble hearing, has a lot of trouble hearing, or deaf). Prior published research has also collapsed these latter variables into a single variable.^16^ Participants were also asked if they currently used a hearing aid.

### Psychological Distress and Health Care Utilization

The Kessler 6 scale was used to evaluate psychological distress.^17^ Participants were asked how much of their time in the past 30 days they felt hopeless, nervous, restless, sad, worthless, or that everything was an effort. Responses are on a graded scale ranging from none (0), a little (1), some (2), most (3), or all of the time (4). Scores are summed to give a total Kessler 6 psychological distress score (range, 0-24). The original proposed cutoff to define severe psychological distress, with probable diagnosable *Diagnostic and Statistical Manual of Mental Disorders* (Fourth Edition) condition(s), was a Kessler 6 score of at least 13, but recent research has recommended a cutoff of 5, which was defined as moderate psychological distress that warrants evaluation and treatment.^18^ Kessler scores were used in 3 analyses, as follows: a binary cutoff of at least 13, a binary cutoff of at least 5, and as a continuous variable. Interviewers also asked participants if they saw or talked to a mental health professional in the past 12 months. A total of 974 participants (3.6%) did not have complete data on the Kessler psychological distress scale.

A random sample of respondents were also asked a series of questions as part of the Adult Functioning and Disability Supplement to the NHIS. Those selected were asked if they used medications for feelings of depression or feelings of worry, nervousness, or anxiety, which were classified as antidepressant or antianxiety medication use, respectively. Approximately half of adults were randomly selected for these questions, yielding an unweighted group of 12 723 participants (49.6%) for antidepressant medication and 12 733 participants (49.6%) for antianxiety medication, respectively. Those with missing medication data were similar to those with medication data, but were less likely to report hypertension (OR, 0.87; 95% CI, 0.81-0.94), and less likely to be African American participants than white participants (OR, 0.87; 95% CI, 0.79-0.97).

### Covariates

Self-reported demographic variables include age, race (white, African American, Asian American, or other), sex, marital status (married, widowed, divorced or separated, or never married), family income (<$50 000, $50 000 to $100 000, >$100 000, or unknown), highest education achieved (<12th grade, 12th grade, 1-3 years of college, or ≥4 years of college), and self-reported health status (excellent, very good, good, fair, or poor). Participants were asked if they had smoked 100 or more cigarettes in their life. A modified Charlson Comorbidity Index was also created using self-reported health conditions, which included 1 point each for asthma, emphysema, cardiac arrest, heart disease, stroke, diabetes, ulcer, liver disease, and renal disease and 2 points for cancer.^19^ Age was not included in the modified Charlson Comorbidity Index because it was included separately in the regression analyses. A total of 163 adults (0.6%) had missing data on comorbidities.

### Statistical Analysis

Analyses were conducted with sample weights provided by the NHIS to be representative of the civilian noninstitutionalized US population. Multiple logistic regression models and Poisson regression models with incident rate ratios were used to evaluate the association between levels of HL and outcomes of interest. Analyses of hearing aid use and outcomes of interest were limited to individuals with moderate or worse HL. Multivariate models included age, sex, race, family income, education, marital status, self-reported health, and cardiovascular comorbidities (ie, hypertension, diabetes, heart disease, stroke, and smoking history). A separate analysis was performed using the modified Charlson Comorbidity Index instead of individual cardiovascular comorbidities. Subgroup analyses were performed using a cutoff of 5 for the Kessler 6 score to assess effect modification by age (ie, <65 years vs ≥65 years) and sex. All analyses were completed using Stata version 13 (StataCorp). Statistical significance was set at *P* < .05, and all tests were 2-tailed.

## Results

A total of 25 665 adults (mean [SD] age, 47.0 [18.1] years, 51.7% [95% CI, 51.0%-52.5%] women, weighted to be representative of the US adult population) were included in the analysis. Baseline weighted population characteristics stratified by self-reported HL are presented in Table 1. Overall, 11 558 participants (49.3%; 95% CI, 48.2%-50.5%) had no HL, 9390 (35.3%; 95% CI, 34.4%-36.2%) had mild HL, and 4717 (15.4%; 95% CI, 14.8%-16.0%) had moderate or worse HL. Self-reported HL was more severe among older participants (eg, age ≥80 years with no HL, 1.6%; 95% CI, 1.4%-1.9%; with mild HL, 4.0%; 95% CI, 3.7%-4.5%; moderate or worse HL, 14.9%; 95% CI, 13.8%-16.1%). Rates of psychological distress, antidepressant medication use, antianxiety medication use, and use of mental health services were higher in those with greater hearing loss (eg, moderate or worse HL vs no HL, moderate psychological distress: 30.3% [95% CI, 28.5%-32.0%] vs 17.1% [95% CI, 16.3%-18.0%]; antidepressant medication use: 15.4% [95% CI, 13.7%-17.2%] vs 5.9% [95% CI, 5.2%-6.6%]; antianxiety medication use: 15.1% [95% CI, 13.5%-17.0%] vs 6.6% [95% CI, 5.8%-7.5%]; use of mental health services: 11.2% [95% CI, 10.2%-12.4%] vs 8.3% [95% CI, 7.6%-8.9%]).

**Table 1.** Demographic Characteristics, Psychological Distress, and Mental Healthcare Utilization by Self-Reported Hearing Category, 2017 National Health Interview Survey^a^

Characteristic	% (95% CI)			
	Total (unweighted n = 25 665)^c^	Hearing loss^b^		
		None (unweighted n = 11 558)	Mild (unweighted n = 9390)	Moderate or worse (unweighted n = 4717)
% (95% CI)	NA	49.3 (48.2-50.5)	35.3 (34.4-36.2)	15.4 (14.8-16.0)
Age, y				
18-29	21.3 (20.5-22.2)	28.6 (27.3-30.0)	17.6 (16.5-18.8)	6.5 (5.4-7.8)
30-39	17.2 (16.6-17.9)	22.8 (21.7-23.9)	14.0 (13.1-14.9)	7.0 (6.1-8.0)
40-49	16.0 (15.4-16.7)	17.9 (17.0-18.9)	15.7 (14.8-16.8)	10.7 (9.6-11.9)
50-59	17.1 (16.4-17.7)	14.3 (13.9-15.2)	20.1 (18.9-21.2)	19.0 (17.4-20.7)
60-69	15.1 (14.6-15.7)	10.1 (9.4-10.7)	18.4 (17.4-19.4)	23.7 (22.2-25.3)
70-79	8.7 (8.3-9.2)	4.7 (4.3-5.1)	10.2 (9.5-10.8)	18.3 (17.0-19.6)
≥80	4.5 (4.3-4.8)	1.6 (1.4-1.9)	4.0 (3.7-4.5)	14.9 (13.8-16.1)
Sex				
Women	51.7 (51.0-52.5)	54.7 (53.5-55.8)	50.4 (49.1-51.7)	45.4 (43.6-47.3)
Men	48.3 (47.5-49.0)	45.3 (44.2-46.5)	49.6 (48.3-50.9)	54.6 (52.7-56.4)
Race				
White	78.0 (76.7-79.3)	74.2 (72.4-75.8)	79.6 (78.1-81.1)	86.9 (85.5-88.2)
African American	12.2 (11.2-13.3)	14.4 (13.1-75.8)	11.6 (10.5-12.9)	6.7 (5.8-7.8)
Asian American	6.3 (5.7-7.0)	8.1 (7.3-9.1)	5.1 (4.4-5.9)	3.2 (2.6-4.0)
Other	3.4 (2.9-4.0)	3.3 (2.7-4.0)	3.7 (3.1-4.4)	3.2 (2.5-4.1)
Annual family income, $				
<50 000	36.9 (35.7-38.1)	35.2 (33.7-36.7)	36.6 (35.1-38.0)	43.0 (41.0-45.0)
50 000-100 000	27.8 (27.0-28.7)	27.7 (26.5-28.9)	28.5 (27.3-29.8)	26.6 (25.0-28.2)
>100 000	27.6 (26.5-28.8)	29.6 (28.1-31.1)	27.0 (25.6-28.5)	22.5 (20.8-24.4)
Unknown	7.7 (7.1-8.3)	7.5 (6.7-8.4)	7.9 (7.1-8.7)	7.9 (6.9-9.1)
Education				
<High school	9.5 (8.9-10.1)	8.3 (7.6-9.1)	10.2 (9.3-11.2)	11.5 (10.2-12.8)
High school	26.2 (25.3-27.1)	24.5 (23.4-25.7)	26.9 (25.7-28.2)	29.8 (28.2-31.5)
Some college	30.3 (29.4-31.3)	29.9 (28.6-31.3)	30.6 (29.3-31.9)	30.8 (29.1-32.6)
≥Bachelor degree	34.0 (32.8-35.3)	37.2 (35.6-38.8)	32.3 (30.7-33.9)	27.9 (25.9-29.9)
Marital status				
Married	52.9 (52.0-53.8)	51.2 (49.8-52.5)	53.9 (52.5-55.3)	56.1 (54.3-57.9)
Widowed	6.0 (5.7-6.3)	3.4 (3.0-3.7)	6.5 (6.1-7.0)	13.1 (12.2-14.1)
Divorced or separated	13.2 (12.7-13.7)	10.8 (10.1-11.5)	14.7 (13.9-15.6)	17.4 (16.2-18.7)
Never married	27.9 (27.0-28.8)	34.7 (33.3-36.1)	24.8 (23.6-26.0)	13.3 (12.0-14.7)
Self-reported health status				
Poor	2.7 (2.5-3.0)	1.4 (1.2-1.7)	2.7 (2.4-3.1)	6.9 (6.1-7.8)
Fair	9.7 (9.2-10.1)	5.9 (5.4-6.4)	11.1 (10.2-12.0)	18.6 (17.2-20.1)
Good	26.6 (25.9-27.3)	21.3 (20.3-22.3)	31.7 (30.5-32.9)	32.0 (30.3-33.7)
Very good	32.8 (32.0-33.6)	32.2 (31.1-33.3)	35.0 (33.8-36.2)	29.6 (27.7-31.5)
Excellent	28.3 (27.4-29.1)	39.3 (38.0-40.5)	19.5 (18.4-20.7)	12.9 (11.7-14.3)
Heart disease	10.1 (9.7-10.7)	6.0 (5.5-6.5)	10.6 (9.9-11.4)	22.4 (21.1-23.8)
Hypertension	30.4 (29.6-31.1)	21.4 (20.5-22.3)	34.2 (33.0-35.4)	50.2 (48.3-52.2)
Diabetes	12.1 (11.6-12.7)	8.1 (7.5-8.8)	13.9 (13.1-14.8)	20.8 (19.4-22.3)
Stroke	3.1 (2.8-3.4)	1.7 (1.4-2.0)	3.1 (2.7-3.6)	7.6 (6.7-8.5)
Smoked 100 cigarettes in lifetime	36.3 (35.4-37.3)	29.3 (28.2-30.4)	39.1 (37.7-40.5)	52.5 (50.8-54.2)
Psychological distress				
Moderate, ie, Kessler 6 score ≥5	21.5 (20.8-22.2)	17.1 (16.3-18.0)	23.9 (22.8-25.0)	30.3 (28.5-32.0)
Serious, ie, Kessler 6 score ≥13	3.4 (3.1-3.7)	2.3 (2.0-2.6)	3.3 (2.9-3.8)	7.0 (6.2-7.9)
Kessler 6 score, mean (range)	2.7 (2.6-2.8)	2.2 (2.1-2.3)	2.9 (2.8-3.0)	3.8 (3.6-3.9)
Taking medication for depression^d^	8.7 (8.0-9.3)	5.9 (5.2-6.6)	9.5 (8.5-10.6)	15.4 (13.7-17.2)
Taking medication for worried, nervous, or anxious feelings^d^	9.2 (8.6-9.9)	6.6 (5.8-7.5)	10.2 (9.1-11.3)	15.1 (13.5-17.0)
Sought mental health care in past year^e^	9.0 (8.6-9.5)	8.3 (7.6-8.9)	9.1 (8.4-9.9)	11.2 (10.2-12.4)
Currently using hearing aids	3.5 (3.2-3.7)	0.09 (0.05-0.15)	0.7 (0.5-0.9)	20.6 (19.2-22.1)

Abbreviation: NA, not applicable.

^a^ Percentages and 95% confidence intervals based on survey weighting provided by National Health Interview Survey.

^b^ Hearing loss categories based on participant self-report of the quality of their hearing without a hearing aid. Responses were collapsed into 3 hearing categories: no hearing loss, response of excellent hearing; mild hearing loss, response of good hearing; and moderate or worse hearing loss, response of has a little trouble hearing, has moderate trouble hearing, has a lot of trouble hearing, or deaf.

^c^ Weighted number of participants was 326 831 120 US adults.

^d^ Approximately half of adults were randomly selected for these questions, yielding an unweighted group of 12 723 and 12 733 participants for antidepressant medication and antianxiety medication, respectively.

^e^ Five participants had missing data for this variable, yielding an unweighted total of 25 660.

Compared with adults without no HL (Table 2), individuals with mild and moderate or worse HL had increased odds of both moderate psychological distress (Kessler score ≥5; mild HL: OR, 1.49; 95% CI, 1.35-1.46; moderate or worse HL: OR, 2.12; 95% CI, 1.87-2.41) and serious psychological distress (Kessler score ≥13, mild HL: OR, 1.16; 95% CI, 0.93-1.45; moderate HL: OR, 2.18; 95% CI, 1.71-2.78) in fully adjusted models. In sensitivity analyses, similar results were observed when investigating the association of HL with the raw Kessler distress score with Poisson regression.

**Table 2.** Association of Self-reported Hearing Loss With Psychological Distress and Medication Use, 2017 National Health Interview Survey^a^

Characteristic	No.	Psychological distress, OR (95% CI)		Kessler raw score, IRR (95% CI)	Medication use, OR (95% CI)^b^		Sought mental health care, past year, OR (95% CI)
		Moderate, ie, Kessler 6 score ≥5	Serious, ie, Kessler 6 score ≥13		Antidepressant	Antianxiety	
All adults							
No hearing loss	11 558	1 [Reference]	1 [Reference]	1 [Reference]	1 [Reference]	1 [Reference]	1 [Reference]
Mild hearing loss	9390	1.49 (1.35-1.64)^c^	1.16 (0.93-1.45)	1.26 (1.20-1.32)^c^	1.39 (1.17-1.67)^d^	1.39 (1.16-1.67)^c^	1.11 (0.98-1.26)
Moderate or worse hearing	4717	2.12 (1.87-2.41)^c^	2.18 (1.71-2.78)^c^	1.59 (1.49-1.69)^c^	2.07 (1.70-2.57)^c^	1.94 (1.57-2.39)^c^	1.53 (1.30-1.79)^c^
Age <65 y							
No hearing loss	9806	1 [Reference]	1 [Reference]	1 [Reference]	1 [Reference]	1 [Reference]	1 [Reference]
Mild hearing loss	6622	1.55 (1.40-1.72)^c^	1.22 (0.97-1.54)	1.28 (1.22-1.35)^c^	1.35 (1.10-1.65)^d^	1.32 (1.07-1.62)^d^	1.07 (0.93-1.23)
Moderate or worse hearing	2247	2.12 (1.83-2.47)^c^	2.25 (1.73-2.93)^c^	1.59 (1.48-1.70)^c^	2.18 (1.69-2.81)^c^	1.91 (1.49-2.44)^c^	1.58 (1.32-1.90)^c^
Age ≥65 y							
No hearing loss	1752	1 [Reference]	1 [Reference]	1 [Reference]	1 [Reference]	1 [Reference]	1 [Reference]
Mild hearing loss	2768	1.10 (0.88-1.37)	0.70 (0.36-1.36)	1.08 (0.95-1.23)	1.62 (1.13-2.32)^d^	2.01 (1.35-2.99)^d^	1.37 (0.98-1.92)
Moderate or worse hearing	2470	1.85 (1.48-2.32)^c^	1.61 (0.84-3.09)	1.49 (1.31-1.70)^c^	2.01 (1.36-2.97)^c^	2.51 (1.66-3.81)^c^	1.50 (1.08-2.08)^e^

Abbreviations: IRR, incidence rate ratio; OR, odds ratio.

^a^ Models adjusted for age, sex, race, family income, education, marital status, self-reported health, heart disease, hypertension, diabetes, stroke, and smoking history. Hearing loss categories were based on participant self-report of the quality of their hearing without a hearing aid. Responses were collapsed into 3 hearing categories: no hearing loss, response of excellent hearing; mild hearing loss, response of good hearing; and moderate or worse hearing loss, response of has a little trouble hearing, has moderate trouble hearing, has a lot of trouble hearing, or deaf.

^b^ Approximately half of respondents were asked about medication use, yielding a total number of participants of 12 723 and 12 733 for antidepressant and antianxiety medications, respectively.

^c^ *P* < .001.

^d^ *P* < .01.

^e^ *P* < .05.

We next investigated whether self-reported hearing loss was also associated with indicators of mental health care utilization (medication use and mental health services). In fully adjusted models (Table 2), there were increased odds of both antidepressant and antianxiety medication use among those with mild HL (antidepressant: OR, 1.39; 95% CI, 1.17-1.67; antianxiety: OR, 1.39; 95% CI, 1.16-1.67) and those with moderate or worse HL (antidepressant: OR, 2.07; 95% CI, 1.70-2.57; antianxiety: OR, 1.94; 95% CI, 1.57-2.39) (Table 2). Compared with individuals without HL, those with moderate or worse hearing loss were also more likely to report seeking mental health care services in the past year (OR, 1.53; 95% CI, 1.30-1.79). Observed associations of hearing loss with mental health care utilization were generally similar in both younger and older adults (<65 years vs ≥65 years) in age-stratified models.

To compare the magnitude of the association of hearing loss and psychological distress with other factors associated with psychological distress, the Figure depicts the associations of other selected covariates from the fully adjusted model with moderate psychological distress. The association of mild HL with moderate psychological distress (OR, 1.49; 95% CI, 1.35-1.64) was greater than associations observed with heart disease (OR, 1.24; 95% CI, 1.09-1.41), hypertension (OR, 1.28; 95% CI, 1.16-1.41), and diabetes (OR, 1.05; 95% CI, 0.93-1.18) and similar to those for history of stroke (OR, 1.51; 95% CI, 1.26-1.81), divorce or separation (OR, 1.41; 95% CI, 1.25-1.58), and family income of less than $50 000 (compared with family income >$100 000; OR, 1.50; 95% CI, 1.31-1.71). The magnitude of the association of moderate or worse HL with moderate psychological distress (OR, 2.13; 95% CI, 1.88-2.42) was greater than ORs observed for any individual cardiovascular risk factor, marital status, or income level.

**Figure.**
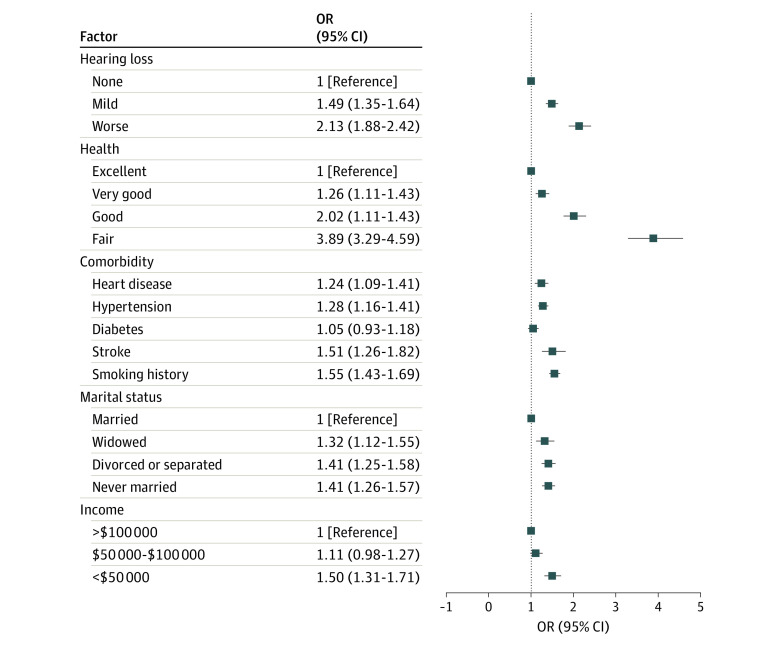
Association of Select Covariates With Moderate Psychological Distress Moderate psychological distress was defined as having a Kessler 6 score of at least 5. OR indicates odds ratio.

We next examined whether self-reported hearing aid use was associated with reduced odds of psychological distress and mental health care utilization (Table 3). Among individuals with moderate or worse HL, we observed that individuals reporting hearing aid use (1066 [22.6%]) vs those who did not (3651 [77.4%]) had a lower odds of moderate psychological distress among all adults (OR, 0.66; 95% CI, 0.53-0.83) and in age-stratified models (Table 3). Hearing aid use was not substantively associated with odds of mental health care utilization except for an observed finding of reduced odds of antianxiety medication use in adults younger than 65 years (OR, 0.35; 95% CI, 0.15-0.83) and increased odds of seeking mental health care in the past year among adults aged 65 years and older (OR, 1.50; 95% CI, 1.01-2.21).

**Table 3.** Association of Hearing Aid Use With Psychological Distress and Medication Use in Individuals With Moderate or Worse Hearing Loss, 2017 National Health Interview Survey^a^

Characteristic	No.	Psychological distress, OR (95% CI)		Kessler raw score, IRR (95% CI)	Medication use, OR (95% CI)^b^		Sought mental health care, past year, OR (95% CI)
		Moderate, ie, Kessler 6 score ≥5	Serious, ie, Kessler 6 score ≥13		Antidepressant	Antianxiety	
All adults							
No hearing aids	3651	1 [Reference]	1 [Reference]	1 [Reference]	1 [Reference]	1 [Reference]	1 [Reference]
Hearing aid use	1066	0.66 (0.53-0.83)^c^	0.73 (0.44-1.22)	0.84 (0.76-0.94)^d^	0.96 (0.63-1.44)	0.72 (0.49-1.07)	1.2 (0.88-1.64)
Age <65 y							
No hearing aids	2030	1 [Reference]	1 [Reference]	1 [Reference]	1 [Reference]	1 [Reference]	1 [Reference]
Hearing aid use	217	0.6 (0.40-0.90)^e^	0.77 (0.37-1.59)	0.86 (0.71-1.04)	0.8 (0.41-1.56)	0.35 (0.15-0.83)^e^	0.91 (0.56-1.48)
Age ≥65 y							
No hearing aids	1621	1 [Reference]	1 [Reference]	1 [Reference]	1 [Reference]	1 [Reference]	1 [Reference]
Hearing aid use	849	0.67 (0.51-0.90)^d^	0.67 (0.32-1.36)	0.83 (0.73-0.95)^d^	0.98 (0.58-1.66)	0.91 (0.56-1.47)	1.5 (1.01-2.21)^e^

Abbreviations: IRR, incidence rate ratio; OR, odds ratio.

^a^ Models adjusted for age, sex, race, family income, education, marital status, self-reported health, heart disease, hypertension, diabetes, stroke, and smoking history.

^b^ Approximately half of respondents asked about medication use, yielding a total number of participants of 2412 and 2419 for antidepressant and antianxiety medications, respectively.

^c^ *P* < .001.

^d^ *P* < .01.

^e^ *P* < .05.

We performed a sensitivity analysis evaluating the raw hearing data (not collapsed in 3 groups) in an adjusted logistic regression model in all ages using the cutoff of 5 for Kessler 6 scores. Compared with those with self-reported excellent hearing, those reporting good hearing (presented elsewhere as having mild HL) had increased odds of psychological distress (OR, 1.49; 95% CI, 1.35-1.64). The other groups also had increased odds of psychological distress (little difficulty hearing: 2886 participants [9.75%; 95% CI, 9.27%-10.25%]; OR, 2.08; 95% CI, 1.81-2.39; moderate difficulty hearing: 1176 participants [3.65%; 95% CI, 3.39%-3.93%]; OR, 2.16; 95% CI, 1.79-2.60; severe difficulty hearing: 579 participants [1.69%; 95% CI, 1.54%-1.86%], OR, 2.44; 95% CI, 1.87-3.19), while those who reported being deaf (76 participants [0.31%; 95% CI, 0.23%-0.41%]) did not have significantly increased odds of psychological distress (OR, 1.90; 95% CI, 0.97-3.71).

We performed additional sensitivity analyses to test the robustness of our observed results. The association between mild HL and a score of at least 5 on the Kessler 6 was significantly lower among those aged 65 years and older compared with those younger than 65 years (OR, 0.70; 95% CI, 0.54-0.90), and there was not a significant difference between age groups for moderate or worse HL (OR, 0.85; 95% CI, 0.65-1.10). Sex-stratified analyses yielded qualitatively similar results for both mild HL (OR, 0.95; 95% CI, 0.78-1.14) and moderate or worse HL (OR, 0.89; 95% CI, 0.72-1.11). Finally, we performed analyses adjusting for a modified Charlson Comorbidity Index rather than adjusting for individual cardiovascular risk factors, and the results did not differ substantively from the presented results (data not shown).

## Discussion

In a large nationally representative sample of US adults, we observed an association between greater self-reported HL and increased odds of psychological distress. Compared with those with no HL, individuals with moderate or greater HL had approximately 2-fold greater odds of reporting psychological distress and using antidepressant or antianxiety medications and approximately 1.5-fold greater odds of seeking mental health care in the past 12 months.

Prior studies have linked HL with a number of adverse health and quality-of-life outcomes. Individuals with impaired hearing have been found to have greater rates of cognitive decline and incident dementia.^20,21,22,23,24,25,26^ The evidence for an association between hearing impairment and depression is mixed. Many larger studies identified increased odds of depressive symptoms with hearing impairment^11,12,13,27,28^; however several smaller studies did not.^29,30^ Several reviews provide a comprehensive analysis and discussion of the existing literature on hearing loss and depression.^5,6,31^ The present study adds to the literature demonstrating the potential association of HL with psychological distress in the largest study, to our knowledge, of a population-representative cohort of noninstitutionalized US adults.

Importantly, findings from the present study demonstrate higher utilization of mental health medications and services in adults with HL. One prior study of 5328 Hispanic adults^14^ found increased rates of depression with HL and a slight increase in the use of antidepressant medications, but we are unaware of other studies demonstrating increased rates of antidepressant or antianxiety medication use or increased utilization of mental health services in adults with HL. Psychological distress and depression are common^1^ and costly to the health care system and economy.^3,4^ Annual US health care expenditures for depression were estimated at $71 billion, the sixth most costly condition, behind diabetes, heart disease, back and neck pain, hypertension, and falls.^32^ Often easily treated with hearing aids or other interventions, HL may be a modifiable risk factor for psychological distress.

The present study found that among those with moderate or worse HL, individuals who reported using hearing aids had lower rates of psychological distress than those who did not. Additionally, adults younger than 65 years with HL who used hearing aids were less likely to use antianxiety medications than those aged 65 years or older. Several prior studies have examined the treatment of hearing impairment and psychiatric outcomes. Analysis of data from 1328 older adults from the Blue Mountain Eye and Hearing Study^33^ found lower rates of depression in individuals with hearing loss who use hearing aids. Pronk et al^34,35^ found a similar result in their cross-sectional analyses of 996 individuals from the Longitudinal Aging Study Amsterdam. In a prospective observational study of 113 individuals with hearing loss receiving hearing aids (n = 63) or cochlear implants (n = 50), loneliness and depression scores improved among those who received cochlear implants at 6 and 12 months and depression scores improved at 6 months among those who received hearing aids.^9,36^ Another study of 94 older adults with profound postlingual deafness^37^ found significantly decreased depression scores at 12 months after cochlear implant.

Rutherford et al^5^ nicely summarized potential mechanisms linking hearing loss and depression in a 2018 review. Effects of HL on social engagement and loneliness as well as on changes in brain structure and function may both contribute to psychological distress and depression. Individuals with HL are more likely to avoid social environments because of difficulty communicating.^38,39^ Several neuroimaging studies have also found decreased activation and decreased volumes in several areas of the brain, including the primary auditory cortex; superior, middle, and inferior temporal gyri; thalamus; brainstem; and parahippocampus,^40,41^ and adults with HL have decreased activation of the parahippocampus, right middle temporal gyrus, and left superior temporal gyrus in response to affective auditory stimuli compared with individuals with no HL.^42^

Our results suggest that hearing impairment may be a strong risk factor for psychological distress, which is reflected in increased rates of utilization of mental health medications and services. Further longitudinal studies and clinical trials are needed to assess the basis of this association and whether treatments for HL could potentially lower rates of psychological distress and utilization of mental health care.

### Limitations

This study has limitations. Our results are from a cross-sectional analysis that limits robust causal inference. While self-reported hearing has been validated as a reliable indicator of hearing measured with criterion-standard audiometry^43,44^ and the prevalence of HL in our study was comparable with prior studies of US adults that used audiometric data,^45,46^ other factors associated with self-assessment of hearing could potentially confound observed associations of HL with psychological distress. We accounted for these potential confounders by adjusting for both self-reported health and cardiovascular risk factors as well as by performing additional analysis with the modified Charlson Comorbidity Index, but residual confounding is still a possibility. Furthermore, our exploratory analyses suggesting that hearing aid use among those with moderate HL may be associated with reduced odds of psychological distress must be interpreted with caution. Individuals with HL using hearing aids vs those who do not use hearing aids are likely to differ across multiple measured (eg, income, education) and unmeasured (eg, health consciousness, health behaviors, family and social status) factors that cannot be fully accounted for in our analyses and may lead to a bias toward seeing beneficial associations with hearing aid use. In contrast, hearing aid use could also be indicative of individuals who are the most severely affected by HL, which could plausibly lead to a potential bias toward seeing hearing aid use as associated with greater psychological distress. We did observe an isolated finding of hearing aid use being associated with greater odds of reporting mental health care utilization by older adults, which is of unclear significance.

## Conclusions

In this study, self-reported HL was associated with psychological distress, antidepressant and antianxiety medication use, and utilization of mental health services in a nationally representative sample of US adults. Further research is warranted to determine whether hearing loss may be a modifiable risk factor for psychological distress.
